# Insight into the Interactions Between GhXI-K and Rab GTPases in Cotton Fiber

**DOI:** 10.3390/plants15030390

**Published:** 2026-01-27

**Authors:** Xinyu Li, Bingke Hao, Junwen Li, Yinhua Jia

**Affiliations:** 1 State Key Laboratory of Cotton Bio-Breeding and Integrated Utilization, Institute of Cotton Research, Chinese Academy of Agricultural Sciences, Anyang 455099, China; 2 National Nanfan Research Institute (Sanya), Chinese Academy of Agricultural Sciences, Sanya 572024, China; 3 Zhengzhou Research Base, National Key Laboratory of Cotton Bio-Breeding and Integrated Utilization, School of Agricultural Sciences, Zhengzhou University, Zhengzhou 450001, China

**Keywords:** cotton, myosin XI-K, Rab GTPases, cell growth protein, polarized growth

## Abstract

Myosin XI-K plays an important role in cell expansion and polarized growth, acting as a motor protein that drives organelle trafficking and cytoplasmic streaming. To elucidate the molecular mechanisms of myosin XI-K’s role in the polarized growth of cotton fiber, we investigated the interactions between GhXI-K and Rab GTPases in cotton (*Gossypium hirsutum*). Protein docking analyses based on AlphaFold3 predicted that GhXI-K interacted with eight Rab GTPases. A total of 37 interaction residues were identified in GhXI-K, of which 5 crucial contact residues were located in the globular tail domain (GTD) and 2 were located in the motor domain. Key interaction residues in the Rab GTPases were also found to be located in conserved regions: switch-I and switch-II. Yeast two-hybrid and bimolecular fluorescence complementation (BiFC) assays confirmed the predictions and showed that these interactions occur primarily in the GTD and the motor domain. Our findings reveal that GhXI-K interacts with Rab GTPases through both the motor and tail domains, suggesting a synergistic mechanism that facilitates polarized vesicle trafficking in cotton fiber cells.

## 1. Introduction

Myosins are well-known motor proteins that drive organelle trafficking and cytoplasmic streaming in plant cells [1]. Specifically, class XI myosins, which are homologous to fungal and animal class V myosins, play crucial roles in cell expansion and polarized growth [2]. Approximately 13 myosin XI genes have been reported in plants, some of which are ubiquitously expressed throughout plant tissues [3]. Among these, myosins XI-K, XI-1, and XI-2 function redundantly in trafficking Golgi stacks, post-Golgi vesicles, mitochondria, peroxisomes, and the endoplasmic reticulum (ER) [4,5,6]. Single and double myosin knockout mutants further demonstrated that XI-K is the predominant driver of cytoplasmic streaming and organelle trafficking [6,7,8,9]. The velocity of cytoplasmic streaming is closely associated with myosin motor activity, which is indispensable for cell and plant growth [10]. Subcellular localization studies have shown that XI-K associates with secretory vesicles and the trans-Golgi network, where it contributes to the exocytosis of cellulose synthase (CESA) complexes during polarized growth [5,11]. This exocytosis relies on the targeted trafficking of secretory vesicles, a process driven by myosin XI-K [12,13].

Rab GTPases play central roles in secretory vesicle trafficking and cellular signaling by directing membrane cargo to the appropriate intracellular destinations [14,15,16,17]. They mediate the selective exchange of proteins, lipids, and CESA cargo between distinct intracellular organelles by recruiting Rab-specific effectors. Beyond organelle trafficking, Rab GTPases also participate in processes such as lysosome exocytosis [18]. For example, Rab3a regulates lysosome positioning and plasma membrane repair [19], whereas Rab11b controls endocytic recycling and secretion [20,21]. The Rab11 subfamily, which includes Rab11a, Rab11b, and Rab25, participates in cytokinesis, ciliogenesis, oogenesis, neuritogenesis, and autophagosome formation [22,23]. However, the role of Rab11 GTPases in lysosome exocytosis remains poorly understood.

Similarly to other small GTPases, Rab11 cycles between active GTP-bound and inactive GDP-bound forms, with GTP hydrolysis facilitated by GTPase-activating proteins (GAPs), and GDP-GTP exchange mediated by guanine nucleotide exchange factors (GEFs) [20]. In its active form, Rab11 recruits effector proteins, including Rab11-family-interacting proteins (Rab11FIPs) [24], motor proteins such as myosin Va/b [25], and the exocyst tethering complex subunit Sec15 [26,27]. Rab11 also interacts with other proteins, such as Rab3A-interacting protein Rabin8 (RAB3IP) [23] and the GEF for Rab3a, GRAB [28].

In plants, polarized delivery of secretory vesicles is a crucial and rate-limiting step for tip growth, and this process depends on myosin XI motor proteins and precise endomembrane regulation [29,30,31]. Rab GTPases contribute to membrane specificity by interacting with a variety of effectors, which are selectively recruited in a nucleotide-dependent manner [32,33]. In yeast, the myosin Myo2 interacts sequentially with Rab GTPases Ypt31/32 and Sec4 to transport vesicles to the growing bud tip and sites of cytokinesis through the transport cascade [34,35,36]. Myo2 and Sec4 interact and localize to secretory vesicles, where they cooperate with the exocyst complex to tether vesicles to the plasma membrane [35,36]. Sec4, a homolog of the Rab8 subfamily in mammals, is known to interact with myosin, and this interaction is conserved across species from yeast to humans [37]. Similarly, mammalian cells exhibit a Rab cascade involved in polarized exocytosis, suggesting that a conserved eukaryotic mechanism underlies this process [16,38]. In this study, we examined the interactions between Rab GTPases and myosin XI-K in cotton to elucidate the molecular mechanisms underlying plant growth and organelle trafficking, shedding light on the conservation of these processes.

## 2. Results

### 2.1. Predicting the Structure of XI-K

**Figure 1:**
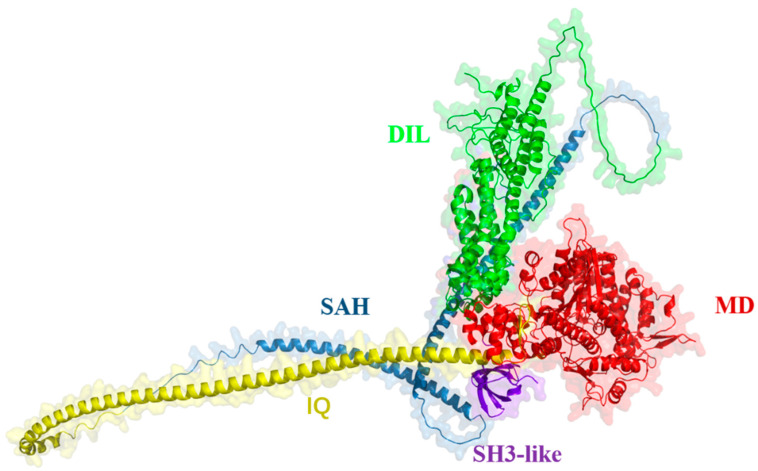
Predicting the structure of GhXI-K with AlphaFold3. MD: motor domain; IQ: neck region; SAH: coiled-coil region; DIL: globular C-terminal tail (GTD); SH3-like: single alpha helix (SAH).

Existing studies have shown that, in plants, myosin consists of four main regions: an N-terminal motor domain (MD), a neck region (IQ), a coiled-coil region, and a globular C-terminal tail (GTD) [39,40]. We found that GhXI-K consists of the four domains described in previous research, identifying an additional SH3-like region at the front end of the MD through the prediction of AlphaFold3 prediction (Figure 1). ATP usually binds to the motor domain, moves a large step along the stiff neck region beside the motor domain, and is released at the end of the reaction [39]. In this hydrolytic cycle, the N-terminal MD also binds to the actin filament, and is released when ATP is moved to the end of the neck region and hydrolyzed to ADP. In the presence of ATP, XI-K usually exists in a stable state with the MD and GTD folded in opposite orientations. The prediction results revealed two loops connecting α-helices and β-sheets in the MD. These two loops might be crucial for protein–protein interactions. The coiled-coil region, composed of two long α-helices, connects the IQ motif to the GTD. The GTD consists of multiple α-helices and loops, and contains the DIL domain, which is responsible for cargo transport (Figure 1).

### 2.2. Predicting the Interactions Between GhXI-K and the Rab GTPases

**Figure 2:**
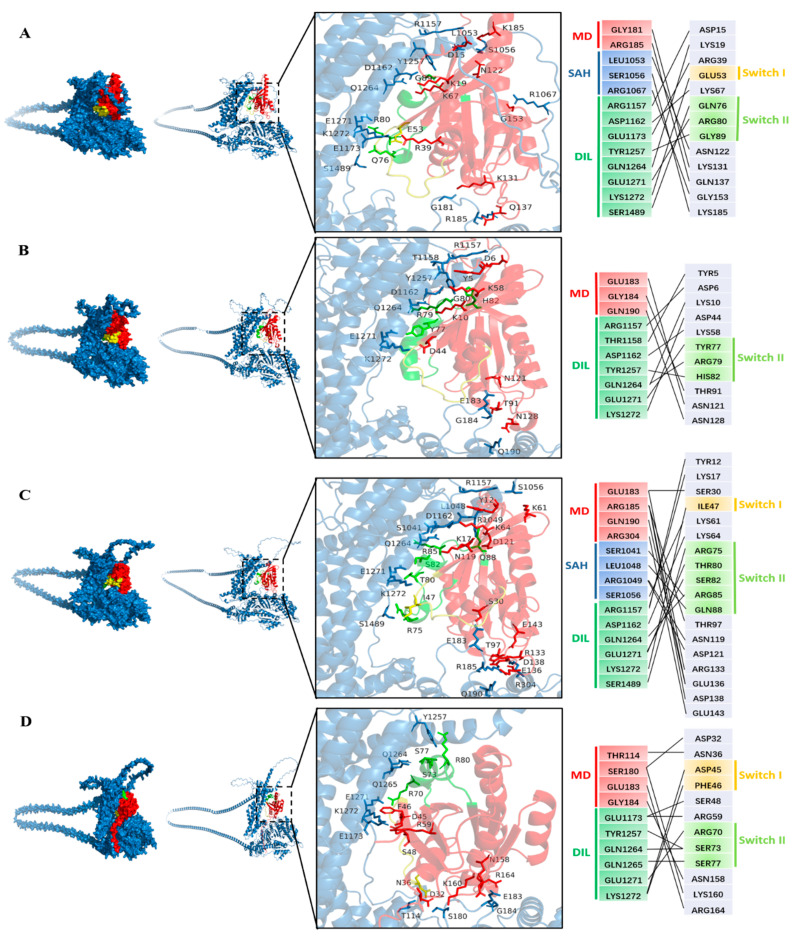

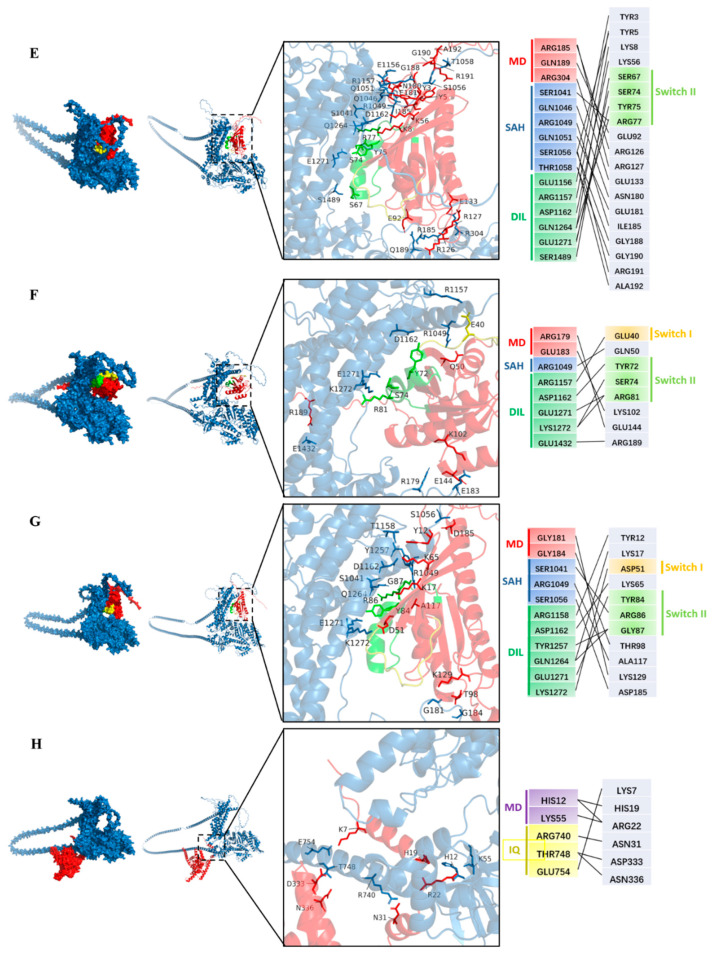
Predicting the interactions of GhXI-K with RabGTPases using AlphaFold3. The color of GhXI-K was blue. The switch I was a yellow color, and switch II was a green color. MD: motor domain; SAH: coiled-coil region; DIL: globular C-terminal tail (GTD). (**A**) GhXI-K interacted with Rab11A. The docking sites of the two proteins are marked in different colors. (**B**). GhXI-K interacted with RabD1. (**C**) GhXI-K interacted with RabC2a. (**D**) GhXI-K interacted with RabH1B. (**E**) GhXI-K interacted with RabB1B. (**F**) GhXI-K interacted with Rab E1C. (**G**) GhXI-K interacted with Rab5. (**H**) GhXI-K interacted with TBC1D2.

Some Rab GTPases, such as Rab-E (Rab8) and Ypt31/32, interact with the myosin XI-K in exocytosis and other processes involving the trafficking of secretory vesicles driven by the myosin motor [31]. These Rab GTPases act as molecular switches and cycle between a GDP “off” state and a GTP-bound “on” state with two conserved motifs named switch-I and switch-II [41]. To screen more Rab GTPases that could interact with GhXI-K in the polar growth of cotton fiber, we investigated nine Rab GTPases, such as GhRab11A, GhRabE1C, GhRabC2a, GhRabD1, GhRabB1B, GhRabH1B, GhRab5, GhRAC7, and GhTBC1D2, which are highly expressed in cotton fiber, according to the cotton genomic database. We predicted the structure of interactions between GhXI-K and the Rab GTPases using AlphaFold3 and analyzed the dock sites. The results revealed that GhXI-K interacted with eight Rab GTPases, including GhRab11A, GhRabE1C, GhRabC2a, GhRabD1, GhRabB1B, GhRabH1B, GhRab5, and GhTBC1D2, at a total of 37 amino acid sites (Figure 2). Among these interaction sites, ARG1157, ASP1162, GLN1264, GLU1271, and LYS1272, located in the GTD, were the key amino acids, which might affect the efficiency of Rab GTPases binding to the GTD. GLU183 and ARG185 were the key amino acids located in the motor domain. These two sites, which are located in the loop connecting the α helix and β sheet, might affect the efficiency of GTP hydrolysis.

Previous studies demonstrated that Rab GTPases such as Rab-E (Rab8) and Ypt31/32 interact with myosin XI during exocytosis and secretory vesicle trafficking [31]. These Rab GTPases act as molecular switches, cycling between a GDP-bound “off” state and a GTP-bound “on” state with two conserved motifs named switch-I and switch-II [41]. To identify more Rab GTPases that could interact with GhXI-K in the polar growth of cotton fiber, we examined nine highly expressed Rab GTPases, such as GhRab11A, GhRabE1C, GhRabC2a, GhRabD1, GhRabB1B, GhRabH1B, GhRab5, GhARAC7, and GhTBC1D2, using protein structure prediction and docking analysis in AlphaFold3. The results revealed that GhXI-K interacted with eight Rab GTPases, excluding GhARAC7, at a total of 37 amino acid sites (Figure 2). Five residues, including ARG1157, ASP1162, GLN1264, GLU1271, and LYS1272 in the GTD, were the key contact points influencing Rab GTPase binding. GLU183 and ARG185 were the key residues located in the loop connecting the α helix and β sheet of the motor domain. These two sites may affect the efficiency of GTP hydrolysis.

Interaction residues in the Rab GTPases were also found to be located in conserved regions. GhRab11A contained 13 interaction residues, of which ASP15, LYS19, GLU53, LYS67, and ARG80 interacted with the key residues in the GTD of GhXI-K (Figure 2). GLU53 was located in the switch-I motif, while LYS67 and ARG80 were located in the switch-II motif. GhRabC2a had 17 interaction residues, among which TYR12, LYS17, ILE47, LYS64, ARG85, THR80, and SER82 interacted with the key residues in the GTD of GhXI-K. ILE47 was located in the switch-I motif, while LYS64, ARG85, THR80, and SER82 were located in the switch-II motif. SER30, THR97, ARG133, GLU136, and ASP138 interacted with the key amino residues in the motor domain of GhXI-K. GhRabD1 contained 11 interaction residues, of which ASP6, LYS10, ASP44, LYS58, ARG79, and TYR77 interacted with the key residues in the GTD of GhXI-K. ASP44 was located in the switch-I motif, while ARG79 and TYR77 were located in the switch-II motif. THR91, ASN121, and ASN128 in the loop interacted with the key residues in the motor domain of GhXI-K. GhRabB1B contained 21 interaction residues, of which TYR5, LYS8, LYS56, ARG77, TYR75, and SER74 interacted with the key residues in the GTD of GhXI-K. LYS56 was located in the switch-I, while ARG77, TYR75, and SER74 were located in switch-II. GhRabH1B interacted with GhXI-K at residues, including PHE46, ASP45, and SER73, and ARG70 interacted with the key amino residues in the GTD of GhXI-K. GhRab5 interacted with GhXI-K at eight residues, including GLU40, TYR72, and SER74, and ARG81 interacted with the key residues in the GTD of GhXI-K. GhTBC1D2 interacted with XI-K at six residues, including ASN31, LYS7, and ASN336, and ASP333 interacted with the residues in the IQ of GhXI-K. GhRabE1C interacted with GhXI-K at eight residues, including GLU40, TYR72, andARG81, and SER74 interacted with the key residues in the GTD of GhXI-K.

### 2.3. Validation of the Interactions Through Yeast Two-Hybrid and BiFC Assays

**Figure 3:**
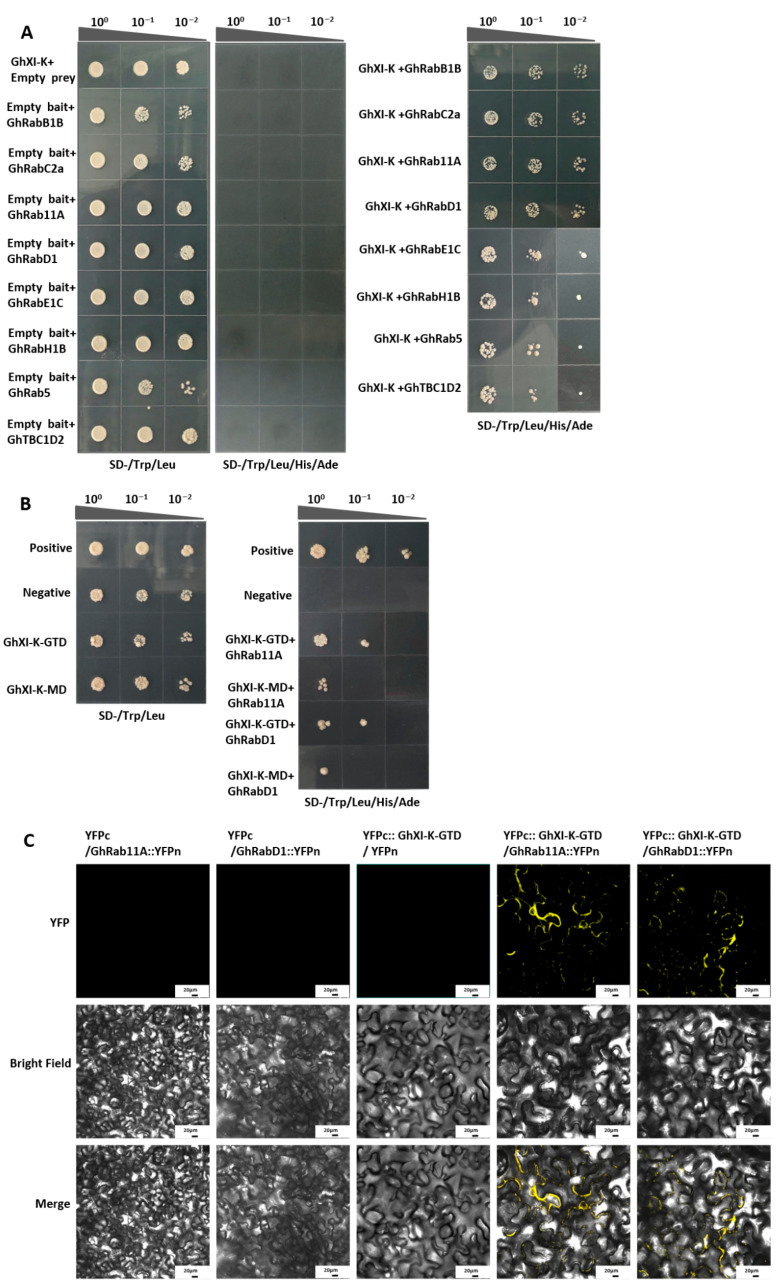
Interaction analysis of GhXI-K-GTD and RabGTPases by yeast two-hybrid assays and bimolecular fluorescence complementation (BiFC). (**A**) Yeast two-hybrid assays between GhXI-K and eight RabGTPases of cotton showed that GhXI-K interacted with GhRab11A, GhRabE1C, GhRabC2a, GhRabD1, GhRabB1B, GhRabH1B, GhRab5, and GhTBC1D2. (**B**) Yeast two-hybrid assays showed that both GhXI-K-GTD and GhXI-K-GTD interacted with GhRab11A and GhRabD1. (**C**) Bimolecular fluorescence complementation (BiFC). Nicotiana benthamiana cells co-infiltrated with YFPc::GhXI-K-GTD/GhRab11A::YFPn and YFPc::GhXI-K-GTD/GhRabD1::YFPn showing reconstitution of the yellow fluorescent protein (YFP) signal in the cytosol. Scale bars are 20 µm.

Yeast two-hybrid assays were performed to validate the predicted interactions. The cDNA of GhXI-K was cloned into the bait vector pGBKT7, and the cDNAs of the eight Rab GTPases were cloned into the prey vector pGADT7. The bait and prey vectors were co-transformed into yeast AH109. The results showed that GhXI-K interacted with GhRab11A, GhRabE1C, GhRabC2a, GhRabD1, GhRabB1B, GhRabH1B, GhRab5, and GhTBC1D2, which corroborated the predictions in AlphaFold3 (Figure 3A). To further confirm domain-specific interactions, the GTD and motor domains of GhXI-K were individually cloned into bait vectors and tested with GhRab11A and GhRabD1. Both domains were found to interact with these two Rab GTPases, indicating that both the GTD and motor domains contribute to polarized cell growth functions (Figure 3B).

Bimolecular fluorescence complementation (BiFC) assays were conducted to confirm the interaction between the GTD of GhXI-K and GhRab11A/GhRabD1. The cDNAs of GhRab11, GhRabD1, and the GTD were cloned into the pENTR-D vector and fused with the N terminus of YFP and the C terminus of YFP, respectively. The recombinant constructs were co-transferred into *Nicotiana benthamiana* cells and incubated. The result showed that fluorescence could be clearly observed at the edge of the cell wall, which further proved that the GTD of GhXI-K interacted with GhRab11 and GhRabD1 (Figure 3C). GhRab11 and GhRabD1 might be functionally located on the cell membrane, affecting the formation of the cell membrane wall, and thus the quality of cotton fiber.

## 3. Discussion

Previous evidence demonstrated that myosin XI-K interacts with Rab-E (Rab8) and Ypt31/32 in the exocytosis and other processes for trafficking of secretory vesicles [31]. Our study systematically identified the Rab GTPase proteins capable of interacting with GhXI-K in cotton. Myosins were assumed to be motor proteins driving organelle trafficking and cytoplasmic streaming in plant cells [1]. Previous studies confirmed that myosins such as XI-K, XI-1, and XI-2 promote polar cell elongation in plants [4,5,6,39,40]. For example, myosin XI was involved in the development of root hairs and trichome morphogenesis, as well as organelle trafficking [6,7,9,31]. The globular C-terminal tail is the primary domain that carries and transports cargos. The conserved DUF motif binds specifically to interacting proteins that might bind to other organelles for the trafficking of vesicles. The GTD of XI binds specifically to Rab-E, thereby promoting the polar elongation of root hairs [31]. We found that the globular tail of GhXI-K binds specifically to not only GhRab-E but also to other Rab GTPases such as GhRab11A, GhRabC2a, GhRabD1, GhRabB1B, GhRabH1B, GhRab5, and GhTBC1D2. Five residues in the GTD, including ARG1157, ASP1162, GLN1264, GLU1271, and LYS1272, were found to be crucial for the interactions. Those crucial residues that were anionic and cationic amino acids might form salt bridges with the crucial residues of Rab GTPases that were cationic and anionic amino acids, to regulate the interactions of GhXI-K with Rab GTPases. GhXI-K interacts with Rab GTPases through its motor domain, and GTD synergistically contributes to the long-distance trafficking of secretory vesicles. Previous studies showed that XI-K interacts with Rab GTPases through the globular tail [25,31]. However, we found that the motor domain of GhXI-K also interacts with the Rab GTPases. The key interaction residues located in the motor domain include GLU183 and ARG185. Deleting the motor domain and GTD significantly weakened the interaction between GhXI-K and the Rab GTPases.

Rab GTPases were found to be molecular switches that cycle between a GDP “off” state and a GTP-bound “on” state [41]. They are lipidated at the C terminus, with geranylgeranyl modifications of C-terminal cysteines mediating their membrane association. Rabs undergo a dynamic GTP-dependent rearrangement of two structural regions, named “switch-I” and “switch-II”, which define their “active” GTP-bound state and effector recognition that typically involves an invariant hydrophobic triad [32,42,43,44]. Our study found that the sites interacting with the key residues of the GTD were mainly located in “switch-I” and “switch-II”, further confirming that Rabs are molecular switches, thereby regulating the myosin–Rab complex formation during polarized vesicle trafficking.

Our research confirmed that GhXI-K interacted with the Rab GTPases in cotton fiber. However, the findings were limited because all those results were based on the predictions and in vitro experiments. Transgenic verification should be carried out to prove the function of those genes in the elongation of cotton fiber.

## 4. Materials and Methods

### 4.1. Plant Materials and Methods

The seeds of *Gossypium hirsutum* cv. TM-1 were germinated and cultivated under greenhouse conditions, with a 28 °C day/22 °C night cycle, 40–60% relative humidity, and a 16 h light/8 h dark cycle with the incandescent. These seedlings were used for RNA extraction. Total RNA was extracted from the seedlings using the FastPure Universal Plant Total RNA Isolation Kit (Vazyme Biotech Co., Ltd., Nanjing, China), and 1 μg of RNA was used for first-strand cDNA synthesis with the Superscript^TM^ First-Strand Synthesis System (Invitrogen, Carlsbad, CA, USA). The reactions were performed using the Roche Light Cycle 480 II instrument (Roche, Basel, 560 Switzerland) programmed as follows: an initial denaturation at 95 °C for 3 min, followed by 40 cycles of 95 °C for 25 s, 56 °C for 30 s, and 72 °C for 30 s.

### 4.2. Prediction of Molecular Structures

The genomic sequence alignments for the GhXI-K and Rab GTPases genes were downloaded from the Gossypium Resource and Network Database (https://cottonfgd.org/ (accessed on 16 March 2022)). Protein sequences were converted based on the sequences of *GhXI-K* and *Rab GTPases*.

The 3D structures of GhXI-K and the GTD were predicted using AlphaFold3 through Google Colab (https://alphafoldserver.com/welcome (accessed on 12 July 2025)) following guidelines in the document [45]. The interactions between GhXI-K and nine Rab GTPase proteins were predicted with AlphaFold3. The visualization and display of modeled structures were executed using the align command in PyMOL (The PyMOL Molecular Graphics System, Version 2.3.5, Schrödinger, LLC., San Diego, CA, USA).

### 4.3. Yeast Two-Hybrid Assay

The cDNAs of eight Rab GTPases were cloned into the prey vector pGADT7. The cDNA encoding GhXI-K, the GTD, and the motor domain were cloned into the bait vector pGBKT7. The bait and prey vectors were co-transformed into the yeast strain Y2HGold. Following growth on SD [DDO for -Trp/-Leu] medium, the transformants were dropped on selective plates [QDO for -Trp/-Leu/-His/-Ade] and maintained at 30 °C for 5 days. The empty bait vector was co-transformed with each prey vector containing the Rab GTPase proteins. AD-T/BD-53 was used as the positive control, and AD-T/BD-Lam was used as the negative control.

### 4.4. Bimolecular Fluorescence Complementation (BiFC) Assay

The cDNA of the GTD fused with the C terminus of YFP was cloned into the pENTR-D vector. The cDNAs GhRab11and GhRabD1 fused with the N terminus of YFP were cloned into the pENTR-D vector. The recombinant constructs were co-transferred into the *Nicotiana benthamiana* cells and incubated. *Nicotiana benthamiana* cells co-infiltrated with YFPc::GhXI-K-GTD/GhRab11::YFPn and YFPc::GhXI-K-GTD/GhRabD1::YFPn showed reconstitution of the yellow fluorescent protein (YFP) signal in the cytosol, while those co-infiltrated with YFPc::GhXI-K-GTD/YFPn, YFPc/GhRab11::YFPn, and YFPc/GhRab D1::YFPn (negative controls) showed no YFP fluorescence. Scale bars are 20 µm.

## 5. Conclusions

This study integrated the interactions of the myosin motor protein GhXI-K with Rab GTPases to reveal a broad and complex regulatory network underlying fiber polarized growth and secretory vesicle trafficking in cotton. Structural prediction results using AlphaFold3 showed that GhXI-K interacted with eight Rab GTPases, including GhRab11A, GhRabE1C, GhRabC2a, GhRabD1, GhRabB1B, GhRabH1B, GhRab5, and GhTBC1D2. Crucial residues that interacted with multiple Rab GTPases were found in both the GTD and the motor domains of GhXI-K. Most of the key residues in the Rab GTPases were located in the conserved switch-I and switch-II motifs. Yeast two-hybrid and BiFC assays further confirmed that the GTD and MD of GhXI-K interacted with GhRab11A and GhRabD1. These findings reveal a conserved eukaryotic strategy in which Rab GTPases and myosin motors cooperate to ensure efficient delivery of secretory vesicles during polarized cell growth, providing a foundation for further investigations of transgenic verification in the regulation of cotton fiber development.

## Data Availability

All data are included in this article.
